# Use of Radiofrequency in Robot-Assisted Partial Nephrectomy for Small Tumors: A Novel Technique

**DOI:** 10.3390/curroncol32050246

**Published:** 2025-04-23

**Authors:** Matías Larrañaga, Helga Ibañez, Jessica Pfeifer, Cristobal Román, Rubén Olivares, José Antonio Salvadó, José Miguel Cabello, Sergio Moreno, Renato Cabello, Carmen Franco, Alfredo Velasco

**Affiliations:** 1 Urology Intern Program, Faculty of Medicine, Finis Terrae University, Santiago 7501014, Chile; mlarranagar@uft.edu; 2 Urology Resident Program, Faculty of Medicine, Finis Terrae University, Santiago 7501014, Chile; hibanezh@uft.edu; 3 Department of Urology, Santa María Clinic, Santiago 7520378, Chile; jpfeifer@clinicasantamaria.cl (J.P.); croman@clinicasantamaria.cl (C.R.); jsalvado@clinicasantamaria.cl (J.A.S.); jcabello@clinicasantamaria.cl (J.M.C.); smoreno@clinicasantamaria.cl (S.M.); rcabello@clinicasantamaria.cl (R.C.); 4 Department of Urology, Cleveland Clinic, Cleveland, OH 44195, USA; olivarr@ccf.org; 5 Department of Pathology, Santa María Clinic, Santiago 7520378, Chile; cfranco@clinicasantamaria.cl

**Keywords:** partial nephrectomy, radiofrequency ablation, tumorectomy, novel technique, nephron-sparing

## Abstract

Introduction and Objectives: Radiofrequency is standardized for ablating small renal tumors, but evidence regarding its effects remains limited. Partial nephrectomy, the gold standard, often leads to hemorrhagic complications and irreversible renal damage due to hilum clamping. To mitigate these risks, we propose a novel technique that replaces clamping with radiofrequency ablation of the tumor for hemostasis in robot-assisted partial nephrectomy. Methods: We report on 357 consecutive patients with T1a renal tumors treated with robot-assisted surgery between 2010 and July 2024. Radiofrequency was used peri-tumorally for hemostasis, followed by complete lesion enucleation. Follow-up included ultrasound and creatinine at 1 month, CT scans at months 3 and 9, and then annually for 5 years. Results: The median age was 60.2 years, with 251 men (70.3%). The median tumor size was 22 mm, and the median blood loss was 15 mL. Hemorrhagic complications occurred in eight patients (2.2%), with one requiring a blood transfusion (0.28%). A total of 30 patients experienced transient stage 1 acute kidney disease (8.4%), with no significant change in median 74.92 mL/min/1.77 m^2^ vs. 78.77 mL/min/1.77 m^2^ vs. (*p*-value 0.15). The median follow-up was 48.2 months, with no tumor recurrence at the treated site. Renal cell carcinoma was found in 83.7% of tumors. Conclusions: To our knowledge, this series represent the largest global undertaking of renal tumor treatment using peripheral radiofrequency ablation without clamping, demonstrating optimal surgical and oncological outcomes, lower morbidity, and fewer complications compared to those noted in the revised literature regarding traditional clamping techniques.

## 1. Introduction and Objectives

The use of radiofrequency has been standardized for the management of solid tumors and metastases [1,2]. In renal cancer, radiofrequency has traditionally been used as an ablation method via CT-guided percutaneous puncture for small lesions or in multiple tumors, such as those occurring in von Hippel–Lindau syndrome.

The 2023 AUA guidelines recommend that clinicians prioritize partial nephrectomy for every patient with cT1a renal masses. Alternatively, radiofrequency ablation (RFA) should be considered in patients who are not candidates for surgery. The guidelines highlight the promising use of cryoablation and radiofrequency ablation in selected cases, which has led to its more widespread adoption [3].

Partial nephrectomy (open, laparoscopic, or robot-assisted) is recognized for producing shorter hospital stays, lower 30-day mortality rates, and better overall survival rates than does radical nephrectomy. However, it carries a higher risk of requiring transfusions and resulting in perioperative bleeding, urinary-source sepsis, acute renal failure, and the need for admission to the intensive care unit, among other risks [4,5,6].

The most common complications associated with laparoscopic or robot-assisted partial nephrectomy include intraoperative and postoperative bleeding, requirement for transfusions, acute kidney injury, and urinary leakage [6,7].

We present this novel technique using radiofrequency for peritumoral hemostasis prior to robot-assisted partial nephrectomy to prevent complications associated with clamping the renal hilum.

### 1.1. History of Radiofrequency Ablation and Applications in Renal Tumors

In 1891, French physicist d’Arsonval reported that the passage of an alternating Radiofrequency current through the liver generates heat without causing neuromuscular excitation. This observation later inspired the development of the Bovie electrosurgical scalpel, which was widely used in operating rooms for over 70 years [8,9,10].

In 1990, McGahan and Rossi were the first to report the use of radiofrequency ablation (RFA) for the treatment of hepatic neoplasms in animal models [11,12]. Since then, RFA has been successfully used to treat osteomas, osteoid osteomas, and small primary and secondary liver tumors [13].

The RFA parameters used for the liver and kidneys are very similar. RFA can be applied using monopolar or bipolar technology, with the monopolar technique being the most used. In this technique, a grounding pad serves as a large dispersive electrode, allowing the electric current to pass through the patient, while the generator activates the electrode to complete the circuit. When the alternating current of 460–500 kHz flows between the electrode and the grounding pad, ionic agitation occurs in the tissue, generating heat through friction. In summary, electrical activation produces kinetic energy and heat, leading to focal thermal injury and tissue necrosis around the tip of the active electrode [14,15,16].

Current radiofrequency generators can automatically adjust the output power to optimize the energy delivered during the ablation process. The higher the current density around the needle electrode, the greater the energy delivered, resulting in a larger ablation area. As Fitzgibbon and Loeser emphasize, “The single most important determinant of tissue heating is RF current density” [15,16,17].

Homeostasis can be maintained when the temperature is elevated to approximately 40 °C. At 46 °C, irreversible cellular damage occurs, but not necrosis; however, when the temperature reaches around 50–52 °C, only 4 to 6 min are needed to induce a cytotoxic effect on tumor cells, resulting in coagulative necrosis and irreversible thermal damage due to the loss of cytosolic and mitochondrial enzymatic activity [17,18].

At temperatures above 60 °C, cell death is almost instantaneous. As the temperature rises, the diameter of the coagulation and necrosis area increases, although this varies depending on the tissue’s conductivity and composition. At temperatures exceeding 105 °C, ablation produces boiling, vaporization, and carbonization. The gas produced acts as an insulator, preventing further energy transmission [18].

A key aspect of RFA is maintaining a temperature between 50–100 °C throughout the tumor volume, without causing gas vaporization.

### 1.2. Rationale and Objective of This Technique

Over the last decade, advances in imaging technology have led to earlier detection of smaller renal masses. More than 75% of these tumors are asymptomatic and incidentally detected through imaging studies, making conservative management the gold standard [19,20,21,22]. Additionally, with an aging population and increasing comorbidities, it is crucial to explore therapeutic alternatives that reduce complications and promote early recovery. Radiofrequency ablation plays a role in managing renal tumors, as it is technically feasible, results in low morbidity, is cost-effective, and offers good oncological and functional outcomes [23,24,25,26,27].

The aim of this study is to describe a modified application of the RFA technique: in a consecutive series of patients with renal tumors, peritumoral hemostatic radiofrequency (PTHR) was applied using robot-assisted techniques. Tumorectomy was conducted once hemostasis was achieved. In this procedure, tumor ablation is not performed; instead, radiofrequency is used as a hemostatic method, allowing enucleation of the lesion without renal hilum clamping.

## 2. Materials and Methods

We present this retrospective descriptive study to share our experience with the PTHR technique. All the surgeries were performed in the operating rooms at Clínica Santa María, Providencia, Santiago, Chile.

The patients eligible for surgery were those diagnosed with a renal tumor exhibiting tomographic characteristics of malignancy and smaller than 4 cm via contrast-enhanced abdominal and pelvic computed tomography or magnetic resonance imaging (CT or MRI).

Patients included in the study were men and women over 18 years old with a confirmed imaging diagnosis of clear cell renal cell carcinoma (RCC) or its variants, based on contrast-enhanced abdominal and pelvic CT or abdominal MRI. Eligible patients displayed at least one exophytic or partially endophytic renal tumor measuring ≤4 cm, regardless of its location, according to computed tomography measurements. Additionally, all participants exhibited an ECOG performance status of 0–1, accepted the risks associated with general anesthesia, and provided informed consent for surgery. Patients were excluded if they showed metastasis at the time of diagnosis, contraindications for general anesthesia, pre-existing heart or pulmonary diseases, or pregnancy. Those with contraindications for imaging follow-up, a history of another metastasized cancer within the last five years, or incomplete medical records were also not eligible for the study.

The main demographic variables measured included age (in years); the presence of type 2 diabetes mellitus, hypertension, or smoking; tumor size in millimeters; the number of tumors and their laterality; tumors in contact with the collecting system; and the reason for consultation prior to undergoing the imaging study.

The intraoperative variables measured comprised the surgical time (in minutes), intraoperative bleeding (in mL), the use or non-use of vascular clamps, and the result of the intraoperative rapid biopsy margins (positive or negative). All the biopsies were reviewed by the same pathologist.

Renal function was measured using the glomerular filtration rate (GFR), according to the MDRD-4 formula in mL/min/1.73 m^2^.

The oncological variables measured included tumor recurrence at the surgical bed and overall mortality.

Finally, the series consists of 357 patients diagnosed with cT1a stage renal tumors identified through CT or MRI who underwent robot-assisted PTHR at Clínica Santa María, Providencia, Chile, between 2010 and June 2024. All patients were treated using the same PTHR technique by a single team led by the same chief surgeon.

All patients were fully informed about the surgery to be performed, the current use of radiofrequency, and the recommendations from the European urology guidelines [28], with the clarification that this surgical technique is a variation of the classic technique recommended by this urology society.

Oncological follow-up was conducted according to the follow-up recommendations outlined in the latest 2023 update of the European Association of Urology (EAU) guidelines on renal cancer [28]. It is important to note that the guidelines stratify follow-up based on risk categories. In this case, our patients fall into the low-risk category (pT1aN0M0), for which the guidelines recommend contrast-enhanced abdominal and pelvic CT scans every 18 months. However, our follow-up protocol aligns more closely with the recommendations for intermediate-risk tumors (pT1bN0). Given that we are evaluating a novel technique, we opted for a more rigorous follow-up strategy.

As part of our protocol, patients underwent contrast-enhanced abdominal and pelvic CT scans at 6 and 12 months postoperatively, followed by annual CT scans until completing five years of follow-up. The only deviation from the guideline recommendations was the addition of an abdominal ultrasound one month after surgery to assess potential local complications, primarily the presence of perirenal hematomas, perirenal fluid collections, or hydronephrosis. Renal function was assessed with serum creatinine one month postoperatively and then annually at each visit. The imaging follow-up was not conducted by the same radiologist.

As this is a retrospective study with data collected over 14 years, we requested a waiver of informed consent for inclusion in the study. Approval for this study was obtained from the ethics committee of Clínica Santa María (ID 190811-24).

### 2.1. Statical Analysis

The normality of the data was assessed using a Shapiro–Wilk test. The median was used as a measure of distribution for all continuous variables. The Mann–Whitney test was used to compare preoperative and postoperative eGFR. A multivariate logistic regression model was used to assess the risk of surgical failure (surgical margins), the risk of bleeding, the risk of a decline in eGFR, and the risk of general complications. To do this, we initially performed a univariate analysis of all the variables. From them, only those variables with a *p*-value < 0.1 were included in the multivariate model. Finally, a statistically significant difference was considered for those variables with a *p*-value < 0.05. All tests were performed using Rstudio software (version 2023.03.0+386). The tables were created using Microsoft Office software (version 16.95.1 (25031528)).

### 2.2. Procedure and Technique

**Figure 1:**
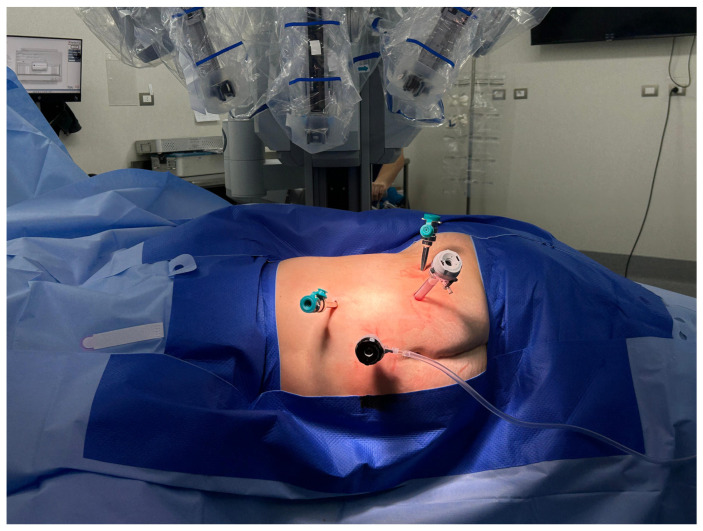
Trocar placement and Da Vinci SI robot docking.

Under general anesthesia, patients were positioned in the lumbotomy position. The trocars were positioned as shown in the image (See Figure 1). The robot was then positioned above the patient’s shoulder, with the camera aligned with the kidney. All surgeries used the Da Vinci SI model.

To access the retroperitoneal cavity the intestine is mobilized to access the kidney. The tumor was then identified using robotic vision by exposing the kidney through the release of the perirenal fat and Gerota’s fascia. For safety, the renal hilum was exposed in all cases to allow for clamping, if necessary.

**Figure 2:**
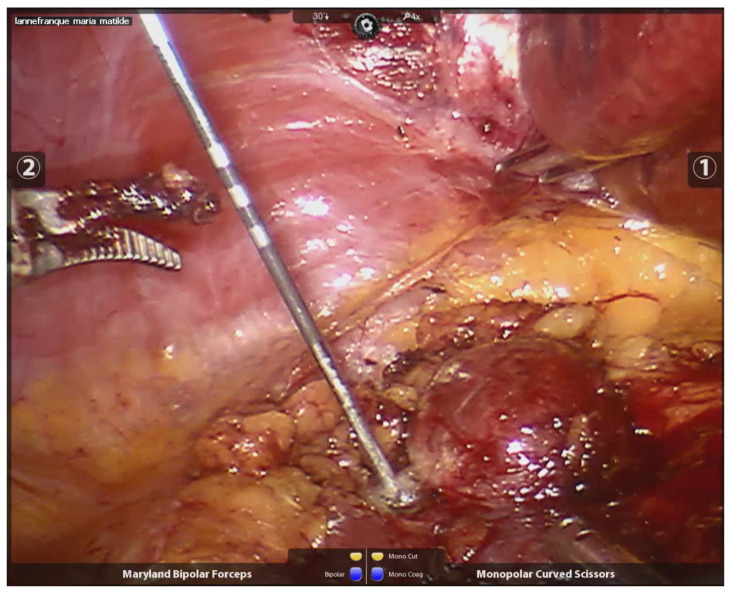
Peritumoral needle positioning and peritumoral radiofrequency.

**Figure 3:**
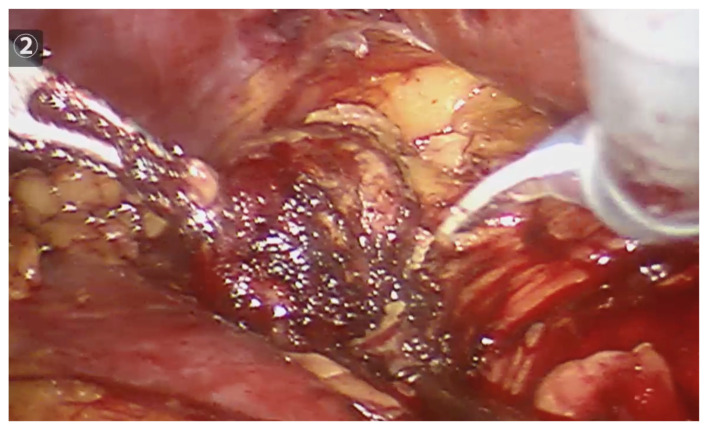
Robotic tumor resection, after completing the peritumoral radiofrequency, clearly shows a necrotic halo, which will guide the resection.

The needle length and diameter were selected based on the size of the tumor and its proximity to or distance from the abdominal wall. The radiofrequency needles had diameters ranging from 1.5 to 3 cm and lengths between 30 and 35 cm. Peri-tumoral radiofrequency needle punctures were performed until maximum impedance was reached, in larger lesions (600–700 kHz), or up to 300–400 kHz, in smaller lesions. The puncture was made 3 mm from the visible tumor edge and 1 to 1.5 cm deep. The needle was not removed until no further evidence of bleeding was observed. The number of punctures varied, depending on the size of the lesion, with all punctures performed peri-tumorally at the border between the tumor tissue and healthy kidney tissue (See Figure 2). After performing radiofrequency along the entire edge of the lesion, a necrotic halo is demarcated, which facilitates its resection (See Figure 3).

**Figure 4:**
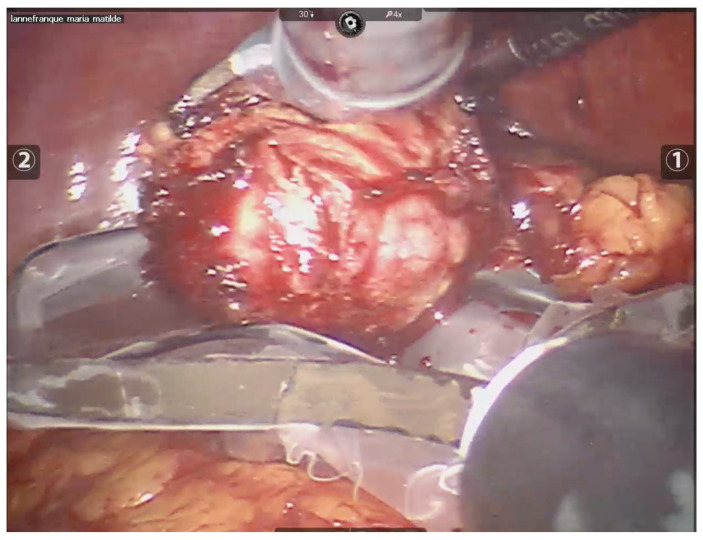
The complete tumor resection is performed, and the tumor’s integrity is preserved, which will facilitate the subsequent biopsy.

**Figure 5:**
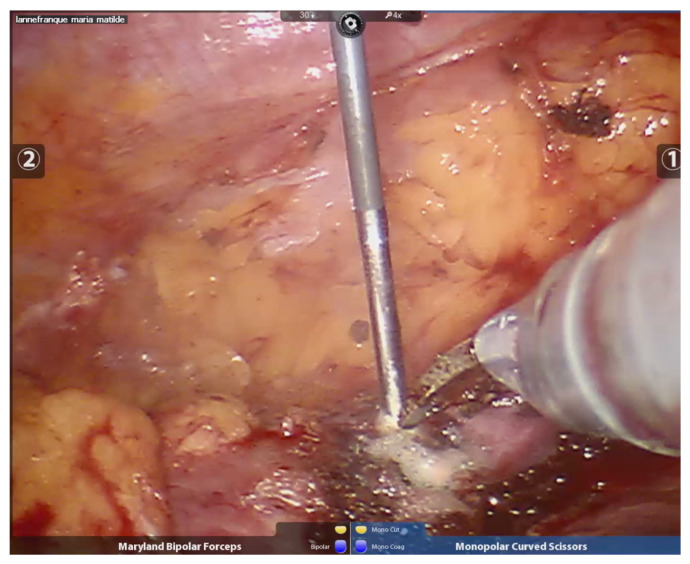
Radiofrequency applied to the surgical bed for hemostatic purposes.

Once peri-tumoral hemostatic control was achieved, the lesion was fully enucleated (See Figure 4). In the case of bleeding during enucleation, surface radiofrequency was applied directly to the bleeding vessel. At the end of the procedure, for oncological safety, radiofrequency was applied to the edges of healthy tissue and to the tumor bed to extend the oncological margin (See Figure 5).

The specimen was always sent for frozen biopsy to confirm complete enucleation and negative margins. Before completing the surgery, superficial radiofrequency (5 mm) was applied to the tumor bed and peri-tumoral edges at 300 kHz for safety, (See Figure 5).

**Figure 6:**
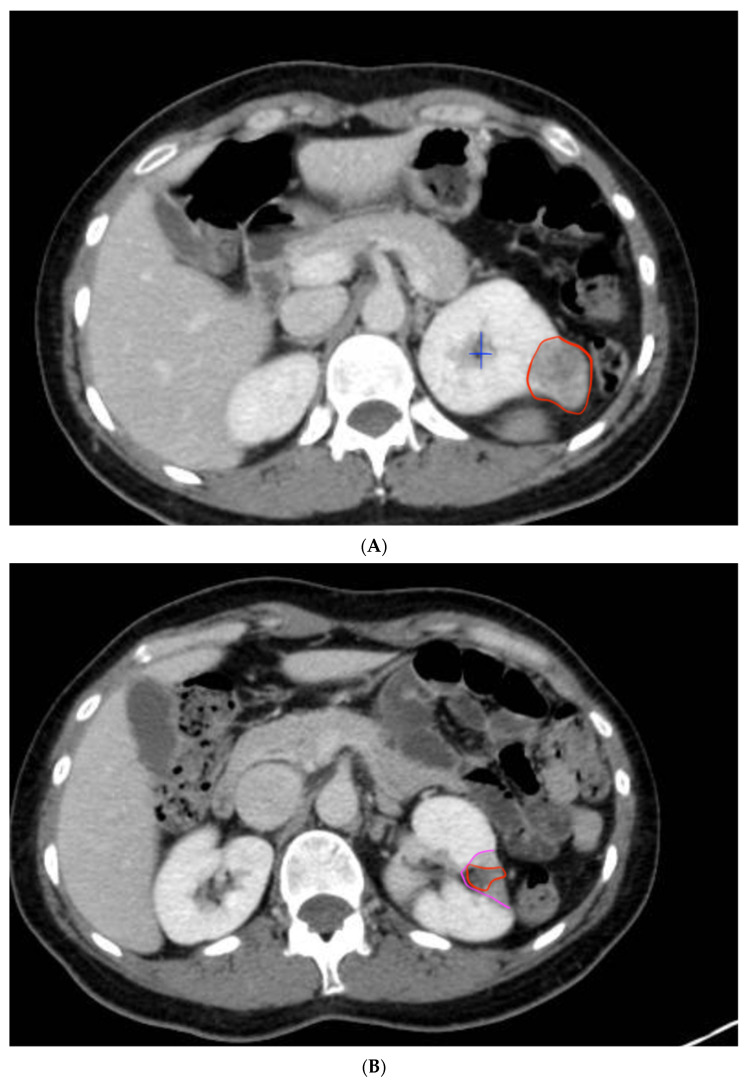
Kidney with exophytic tumor and control CT one year after surgery. (**A**) The first image shows a renal tumor on the posterior surface of the left kidney, measuring 3 cm, with characteristics of clear cell renal carcinoma (encircled in red). It is partially exophytic, located away from the renal hilum and the urinary collecting system. The blue cross marks the renal collecting system. (**B**) The second image shows the absence of the previously described mass. Post-operative changes are evident, with a scar in the area where the lesion was previously located and the morphological alterations of the kidney. Encircled in pink, the peritumoral necrotic halo is visible. In red, the bed where the tumor was located can be seen.

The persistent absence of contrast, hypervascularization, or vascular necrosis in the peri-tumoral area in the control CT was considered indicative of successful ablation, without recurrence (See Figure 6A,B).

## 3. Results

The median age was 60.2 years (IQR 52–70), and 251 (70.3%) of the patients were men. A total of 345 patients displayed a single tumor (96.6%), while 12 had two or more tumors (3.3%). Most of the tumors were left-sided (54.2%), and the median tumor size was 22 mm (IQR 12–31). The demographic and preoperative information is described in Table 1.

The median surgical time was 115 min (IQR 90–140). The median blood loss was 15 mL (IQR 10–40). The number of peritumoral punctures performed ranged from 8 to 14 and was directly related to the size of the tumor (median of 9).

A rapid frozen section biopsy was routinely performed in all patients, and positive intra-operative margins were reported in seven cases (1.96%). In 83.7% of the patients, the final diagnosis was renal cell carcinoma (RCC) (n = 306), 7% showed papillary tumors (n = 26), 4.6% displayed angiomyolipomas (not suspected on CT or MRI prior to surgery) (n = 17); oncocytoma was diagnosed in 7 cases (1.8%), and 18 were cysts (4.8%).

All procedures were completed, and none required conversion to open surgery. However, in three of these procedures, vascular clamping was necessary. The first two correspond to the initial procedures performed. In the other case, it was necessary to clamp a segmental renal artery due to persistent bleeding.

Following surgery, patients had a median hospital stay of 54 h (IQR 36–60 h).

The comparison between the median preoperative eGFR and postoperative eGFR was 74.92 mL/min/1.77 m^2^ vs. 78.77 mL/min/1.77 m^2^ vs. (*p*-value 0.15). A total of 19 patients (5.3%) exhibited transient acute renal failure, according to the RIFLE criteria.

The median follow-up was 48.2 (24–58) months. All tumors were found to be well-controlled in the CT follow-up, with none showing contrast enhancement in the tumor area. Three patients developed new renal tumors; however, none were considered recurrences due to their location: two were in the opposite pole of the same kidney, and one was in the contralateral kidney.

No patients developed metastases during follow-up, and no deaths occurred during the specified period.

Of the 357 patients, 92 have not yet completed their recommended 5-year post-surgery follow-up period, according to EUA guidelines. However, there are 186 patients in our series who, by personal preference, decided to continue follow-up and have undergone at least one imaging control procedure after completing the recommended follow-up period, with a median follow-up of 93.6 months, without showing any metastasis or tumor recurrence at the surgical bed.

We used a logistic regression model to predict surgical failure (defined as positive surgical margins), intraoperative bleeding, the risk of worsening eGFR, and the overall risk of complications. The results are presented in Table 2:

### 3.1. Hemorrhagic Complications

In the entire series, blood transfusion was required in one patient (0.28%), who had undergone a previous contralateral radical nephrectomy and presented with a low preoperative basal hematocrit count. Hemorrhage occurred in seven patients (1.96%), either intraoperatively (3) or postoperatively (4). A reoperation was necessary due to persistent bleeding from an epigastric artery at one of the robotic trocar puncture sites, with no bleeding at the renal surgical site.

### 3.2. Urinary Leakage or Fistula

We had one incidence of urine leakage or urinary fistula, or arteriovenous fistula in the immediate or late follow-up of this series (0.28%).

### 3.3. Other Urological Complications

One patient presented moderate hydronephrosis, with possible ureteropelvic stenosis attributable to heat fibrosis, which has been kept under observation, without progression of hydronephrosis; one patient presented a urinary retention due to BPH; three patients presented with hematuria at the 30 days control; and one presented with pyelonephritis.

See Table 3 for the details of the complications, according to the Clavien–Dindo classification system.

## 4. Discussion

The increased use of ultrasound, CT, and MRI for abdominal imaging has led to the incidental detection of more renal tumors. Partial nephrectomy (PN) is the gold standard in the management of stage I renal tumors [3,28,29]. However, the morbidity associated with this technique is directly related to its complexity. Using percutaneous techniques under intra-abdominal vision enables the treatment of exophytic lesions, with a significant reduction in common complications, such as urinary fistulas and intra- and post-operative bleeding.

Intra-operative and post-operative bleeding is not an infrequent complication, reported in up to 9% of cases [6,7,30]. The need for arterial clamping often leads to transient renal function impairment, but prolonged warm ischemia can cause permanent damage [31,32]. While complications from renal pedicle dissection are infrequent, they can be serious and may result in loss of renal units or significant bleeding.

The technique presented here does not require arterial clamping and has not resulted in significant bleeding. PTHR does not preclude the option of conversion to open surgery or a standard laparoscopic PN with clamping, if necessary.

In our series, perioperative bleeding occurred in 1.96% of cases. As the objective of this study, we recorded only three intraoperative complications: all were hemorrhages, with renal artery clamping required in two patients and clamping of segmentary branches in one patient. The local control and ischemia produced by radiofrequency allow for the creation of a peri-tumoral fibrous tissue that prevents late complications. In case of bleeding during enucleation, surface radiofrequency was performed directly on the bleeding vessel. In our series, except for in the first 15 cases, surgical bed drainage was not used.

Urinary leaks ranging from 1.47% to 3.6%, according to some series, are reported in robotic or laparoscopic partial nephrectomies [6,7,30]. In our series, the incidence was 0.28%. In the cases were that the tumor was close to the urinary tract before radiofrequency (n = 3), a ureteral catheter was installed in the renal pelvis before RFA, allowing for the irrigation with cold solution during the procedure to avoid necrosis of the urinary tract and subsequent urinary fistula. One patient presented moderate hydronephrosis, with possible ureteropelvic stenosis attributable to heat fibrosis, that has been kept under observation without progression of hydronephrosis.

The local control and the ischemia produced by radiofrequency allows for the creation of a peritumoral-resistant fibrous tissue that prevents these late complications.

Additionally, a large series involving 1893 patients undergoing partial nephrectomy showed acute renal failure rates of up to 20%, according to the RIFLE criteria [33]. In our series, the acute kidney injury (AKI) rate was 5.3%, and all cases were transient.

The rate of positive surgical margins reported in the literature for partial nephrectomy ranges from 0.1% to 10.7% [34], and in our series, it was 1.96%. In our series, there were three tumor recurrences: two in the same kidney but at a different site, and one in the contralateral kidney. The PTHR technique creates an avascular plane between the tumor and the necrotic renal tissue, which is easily identifiable and facilitates successful tumorectomy. Undoubtedly, the ischemic effect produced by the radiofrequency needle, combined with the radiofrequency applied to the tumor bed and peri-tumoral area at the end of the procedure, contributed to these results. These results demonstrate similar values when compared to those in the literature. In a 20-year follow-up study on radiofrequency ablation for small renal masses, among the evaluated patients, 11 (4.9%) developed tumor recurrence in kidneys away from the ablation zone, while local recurrence in the ablation zone was identified in 7 patients (3.1%). In the present study, the median overall survival (OS) was reported to be 8.8 years [35]. We did not record any deaths during the study period.

Upon reviewing our multivariate logistic regression predictive model, it can be observed that the main predictors of intraoperative bleeding are tumor size, measured on the preoperative abdominal and pelvic scan (*p*-value 0.1), as well as the duration of the surgery (*p*-value 0.05). The latter is believed to be primarily because prolonged surgery is often caused by intraoperative bleeding, which requires more time to control. The only variable for worsening renal function in this study was the preoperative glomerular filtration rate (GFR) value; we believe it is important to complement preoperative studies to determine the origin of previous renal failure, even with the use of renal scintigraphy, to prevent declines in renal function. However, in our series, there was no decline in median postoperative glomerular filtration. Anecdotally, it was found that the previous value of GFR is a predictor of positive surgical margins, but we do not have a clinical explanation for this. Lastly, there was no statistically significant factor to predict the overall presence of complications. However, longer hospital stays could be an indicator of this (*p*-value 0.05).

Our initial experiences with RFA involved patients with bilateral tumors, a case of metastatic melanoma, and other renal tumors. We biopsied the lesions using a Tru-Cut biopsy needle and applied at least one cycle of RFA or until maximum impedance was achieved, resulting in tumor necrosis. No significant artifacts were generated by the RF, allowing for a straightforward microscopic examination.

When comparing the results of this technique with those reported for radiofrequency ablation alone, we found that oncological and perioperative outcomes are inconsistent. Some authors have reported similar overall survival and cancer-specific survival, as well as comparable local recurrence rates when comparing partial nephrectomy with radiofrequency ablation [36,37,38]. Other authors suggest that local recurrence rates and overall survival are worse [39,40]. These inconsistencies mainly arise because there are no clear criteria for performing radiofrequency ablation. Some authors suggest that the indications for radiofrequency ablation should be the same as those for partial nephrectomy, according to the “RENAL score”. However, there are no high-quality recommendations for these indications, and currently, radiofrequency ablation is only indicated when surgery is technically unfeasible due to patient comorbidities, tumor characteristics, or the inability to maintain anesthesia during surgery [41]. A recent systematic review from February 2025 analyzed the recurrence rates of various ablative therapies for renal tumors. The results showed that for radiofrequency ablation, the global local recurrence rate was 4% at one year, 5% at two years, and 8% at five years. For cryoablation, local recurrence was 5% at one year, 6% at two years, and 10% at five years. Microwave ablation recorded a five-year recurrence rate of 14% [42]. Regarding complications, studies conclude that the rate of Clavien–Dindo grade 3 complications reported for radiofrequency ablation is approximately 4%. Hemorrhagic complications have been reported in up to 5% of cases. The main limitations of this technique include the fact that persistent bleeding can only be controlled through arterial embolization, which may be associated with severe complications such as partial or total loss of renal function in the treated unit. Additionally, these techniques do not allow for intraoperative biopsy confirmation or margin assessment after the procedure [35,43].

While the clinical application of PTHR has expanded, this technique will only favorably compare with radical nephrectomy as an acceptable alternative for local cancer control.

Small tumors can be completely excised, and the complication rate remains low when RFA is employed.

The combined approach of treatment with peri-tumoral hemostatic radiofrequency and tumorectomy results in low morbidity, few complications, and short surgical times and post-operative hospital stays.

Our findings suggest that the surgical and oncological results are optimal and comparable with those for the reported series of partial nephrectomy with clamping and ablative therapies alone [34,35,36,37,38,39,40,41,42,43].

## 5. Conclusions

To our knowledge, this is the only published series on the use of peritumoral radiofrequency in robot-assisted partial nephrectomies.

Radiofrequency has proven to be a safe and effective hemostatic technique, providing coagulation and sealing of vessels, lymphatics, and minor urinary tracts. With proper patient selection, most complications associated with nephron-sparing surgery can be avoided.

The tumorectomy with ischemic ablation we performed is a simple and reproducible technique.

In summary, we believe that radiofrequency, with robotic assistance, is a minimally invasive and novel treatment option for preserving renal parenchyma in the management of small renal tumors. Our initial experience demonstrates that this treatment modality is both safe and effective when comparing our results with those reported in the literature for classic partial nephrectomy.

## 6. Limitations of the Study

One of the main limitations of this study is the lack of renal scintigraphy for the patients, which we believe is the optimal method to accurately assess changes in renal function after surgery. Moreover, as this is a descriptive and retrospective study, we believe that the absence of a control group could affect the interpretation of the results.

Unfortunately, therapies such as cryoablation or radiofrequency are not performed alone in our center, making it impossible to conduct a retrospective study of this nature. However, we plan to carry out this study as a clinical trial to compare the oncological and surgical outcomes of this technique with those of traditional partial nephrectomy, both performed with robotic assistance.

We believe that future studies comparing this technique with radiofrequency alone and/or standard surgical techniques would be useful for providing higher-quality conclusions. We are currently involved in this research.

## Figures and Tables

**Table 1:** Summary of presurgical information.

Variant	Value
Median age (IQR)	60.2 (52–70)
No. males (%)	251 (70.3)
No. former or current smokers (%)	169 (47.3)
No. asymptomatic at presentation, image finding (%)	332 (92.9)
No. with hypertension (%)	112 (31.3)
No. with diabetes mellitus (%)	37 (10.3)
No. with solitary kidney (%)	10 (2.8)
No. with prior contralateral PN (%)	5(1.4)
No. right side tumors (%)	232 (45.8)
No. central tumors (abuts collecting system) (%)	15 (4.2)
No. interpolar tumors (%)	92 (25.7)
No. of tumors:	
1	345 (96.6)
2	12 (3.3)
3 or more	0

**Table 2:** Multivariant logistic regression model to predict intraoperative bleeding, complications, and surgical success.

Risk of Intraoperative Bleeding		
Variable	OR (CI 95%)	*p*-Value
Tumor size	3.79 (0.75–19.1)	0.1
Operative time	1.67 (0.98–2.8)	0.05
**Risk of positive margins**		
ASA score	1.05 (0.9–1.23)	0.26
Pre-operative eGFR	0.99 (0.99–0.997)	<0.01
Post-operative eGFR	0.99 (0.99–1)	0.52
**Risk of eGFR decrease**		
Pre-operative eGFR	1.46 (1.23–1.73)	<0.01
**General risk of complications**		
Diabetes mellitus	1.2 (0.97–1.59)	0.09
Hospital stay	0.91 (0.84–0.99)	0.05
Pre-operative eGFR	0.99 (0.99–1)	0.36
Post-operative eGFR	0.99 (0.99–1)	0.71

**Table 3:** Post-operative complications by Clavien–Dindo grading system.

Grade (Organ System)	Complications (No.)	Total No.
I:		9
Genitourinary	Pyelonephritis (1) Hematuria (3)	4
Pulmonary	Atelectasis (1) Nosocomial pneumonia (1)	2
Gastrointestinal	Ileus (1)	1
Dermatological	Rash (1)	1
Other	Pain (1)	1
II:		3
Cardiovascular	Hypertensive crisis (1) Blood transfusion (1)	2
Gastrointestinal	Rectus hematoma (1)	1
III:		7
Genitourinary	Perirenal hematoma (2) Urinary leak (1) Ureteropielyc stenosis (1)	4
Other	Trocar site hernia (3)	3
IV:		1
Cardiovascular	Acute myocardial infarction (1)	1
Total		20

## Data Availability

The data presented in this study are available in this article.
